# Genetic medicine is accelerating in Japan

**DOI:** 10.1007/s12282-022-01342-4

**Published:** 2022-02-21

**Authors:** Saori Hayashi, Makoto Kubo, Kazuhisa Kaneshiro, Masaya Kai, Mai Yamada, Takafumi Morisaki, Yuka Takao, Akiko Shimazaki, Sawako Shikada, Masafumi Nakamura

**Affiliations:** 1 Department of Surgery and Oncology, Graduate School of Medical Sciences, Kyushu University, 3-1-1 Maidashi Higashi-ku, Fukuoka, 812-8582 Japan; 2 Department of Clinical Genetics and Medicine, Kyushu University Hospital, Fukuoka, Japan

**Keywords:** BRCA, Hereditary breast and ovarian cancer syndrome, Genetic testing, Genetic medicine, Olaparib

## Abstract

**Background:**

In 2018, BRACAnalysis® was covered by medical insurance in Japan as a companion diagnostic test for the poly ADP-ribose polymerase inhibitor olaparib. In April 2020, eligibility for *BRCA1/2* genetic testing was expanded to the diagnosis of hereditary breast and ovarian cancer syndrome, and medical management including prophylactic surgery and surveillance were covered by public insurance for *BRCA1/2* mutation carriers who developed breast or ovarian cancer. The amount of *BRCA1/2* genetic testing has been increasing recently, but the number of subjects and the impact of testing for patients’ outcomes remain unclear.

**Patients and methods:**

This study explored the potential number of patients who will be eligible for new insurance coverage for *BRCA1/2* genetic testing. We analyzed 868 patients from 938 surgeries between January 2014 and September 2020 from our database.

**Results:**

Overall, 372 patients (43%) were eligible for new insurance coverage for *BRCA1/2* genetic testing. The most common category was family history of breast or ovarian cancer within third-degree relatives. We found that 202 patients (23%) had family history of breast or ovarian cancer. In addition, the progression-free survival was significantly lower in triple-negative breast cancer patients aged 60 years or younger compared with the other patients (*P* = 0.0005).

**Conclusion:**

The genetic medicine for primary breast cancer patients with *BRCA1/2* germline mutation is accelerating rapidly in Japan. Therefore, establishing a system for the genetic medicine would be urgent.

**Supplementary Information:**

The online version contains supplementary material available at 10.1007/s12282-022-01342-4.

## Introduction

Approximately 5–10% of all breast cancers were reported to be inherited [1–3]. Since the identification of *BRCA1* in 1994 [4] and *BRCA2* in 1995 [5], various studies of hereditary breast and ovarian cancer (HBOC) have progressed rapidly. BRCA protein is important for many functions including DNA repair, transcription, and cell cycle control. Mutations in the *BRCA* gene result in a loss of these functions [6, 7]. However, a previous study reported no significant differences in OS or distant disease-free survival between breast cancer patients with or without *BRCA1/2* mutations [8, 9].

Genetic testing has been popular overseas, particularly in USA, for about 2 decades [10]. Although *BRCA1/2* mutation was reported to be more frequent in Japan than USA and Europe [2], genetic testing has been uncommon until these days. However, since 2018, the use of BRACAnalysis® (Myriad Genetics, Inc., Salt Lake City, UT, USA) has been covered by public medical insurance in Japan as a companion diagnostic test (CDx) for the poly ADP-ribose polymerase (PARP) inhibitor olaparib. In Japan, it was approved by public medical insurance as maintenance treatment of platinum sensitive ovarian cancer in January 2018. Then, based on the results of OlympiAD trial [7], the indication for olaparib was expanded to include patients with germline BRCA mutated and HER2-negative inoperable or recurrent breast cancer who had received prior chemotherapy in July 2018 [11]. BRACAnalysis® was the first CDx for the presence of germline, rather than somatic, pathogenic or likely pathogenic mutations. As a result, fundamental issues such as the establishment of a genetic counseling system and management for VUS have been apparent. In addition, advances in Next-Generation Sequencing have led to the development of comprehensive genomic profiling test and its widespread use in clinical settings; thus, increasing the opportunity to detect *BRCA1/2* mutations as secondary findings [12].

In April 2020, Japanese national insurance coverage was extended to *BRCA1/2* genetic testing for suspected HBOC based on the following six criteria including onset at age 45 years or younger; triple-negative breast cancer at age 60 years or younger; two or more primary breast cancers; history of ovarian, third-degree relatives with breast or ovarian cancer; history of ovarian, fallopian tube, or peritoneal cancer; or male breast cancer. It also covered risk-reducing mastectomy (RRM), risk-reducing salpingo-oophorectomy (RRSO), reconstructive surgery, and surveillance for breast or ovarian cancer patients carrying a *BRCA1/2* pathogenic or likely pathogenic mutation.

This revision of public insurance will reduce the financial burden on and will benefit medical management of breast cancer patients who have been unable to choose genetic testing or prophylactic treatment. It was previously reported that patients who undergo prophylactic resection are more likely to have a reduced psychological burden related to a fear of cancer, although they are still affected by changes in body image, menopausal symptoms, and sexual well-being [13]. To date, risk-reducing surgery is less common in Japan than in USA and Europe [2, 14]. The amount of *BRCA1/2* genetic testing, RRM, RRSO, reconstructive surgery, and surveillance are predicted to increase economic and human resources burden in coming years. Our study aimed to analyze the number of subjects and the impact of testing for patients’ outcomes.

## Patients and methods

### Patients

The aim of this study was to explore the potential number and outcomes of patients who will be eligible for *BRCA1/2* genetic testing under the new medical insurance coverage. New categories included in the medical insurance are as follows: onset at age 45 years or younger; triple-negative breast cancer at age 60 years or younger; two or more primary breast cancers; a history of ovarian, third-degree relatives with breast or ovarian cancer; a history of ovarian, fallopian tube, or peritoneal cancer; or male breast cancer. We surveyed our database that included 938 breast cancer patients with radical surgeries performed in our department between January 2014 and September 2020. The average follow-up duration was 24.3 months. Age, sex, family history of cancer within third-degree relatives, history of cancer, menopausal status, preoperative treatment, TNM stage classification, hormone receptor (HR) status, HER2 status, and postoperative treatment were recorded in the database. Moreover, we assessed our data to search for patients with suspected HBOC for genetic testing by BRACAnalysis®, which was newly covered by the Japanese public insurance from April 2020.

**Fig. 1:**
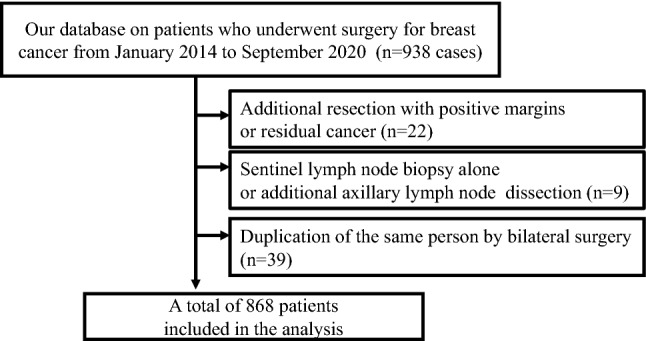
Flowchart of patient selection

Of the 938 patients, 868 were evaluated, excluding duplication by synchronous bilateral breast cancer and additional resection with positive margins or residual cancer, and reoperation of the axillary lymph nodes such as sentinel lymph-node biopsy and axillary lymph-node dissection (Fig. 1). The characteristics of the patients are shown in Table 1. Of the 868 patients, all were female except for one male. The age of all patients ranged from 26 to 92 years, with a median of 60 years. 43% (386/868) patients had stage I and the stage classification was counted per breast. Of the 868 patients, 39 individuals underwent bilateral breast surgery during the study period, so the stage and subtype category were evaluated based on 907 breasts. Stage 0 included 115 patients of DCIS and 5 with Paget’s disease. Subtype classification was evaluated in 787 patients, excluding 120 patients at stage 0.70% (637/787) patients had HR-positive disease. However, specimens of patients who achieved a pathological complete response (pCR), or those who had small lesions that were difficult to assess after neoadjuvant chemotherapy (NAC) were evaluated using preoperative core needle biopsy samples. We defined two or more primary breast cancers as synchronous bilateral breast cancer, metachronous bilateral breast cancer, or metachronous ipsilateral breast cancer with no apparent intramammary recurrence, such as recurrence near the surgical margin after partial breast resection.

**Table 1:** Patient characteristics

Age		
Range (median), years	26–92 (60)	
Sex	Number of patients	%
Female	867	99.9
Male	1	0.1
pStage		
0	121	13
DISC	116	
Paget’s disease	5	
I	386	43
IIA	187	21
IIB	106	12
IIIA	41	5
IIIB	14	2
IIIC	15	2
IV	12	1
Unknown	26	3
Total	868	100
Subtype	Number of breasts	
HR-positive		
Luminal A/B	552	60.9
Luminal HER2	84	9.3
HR-negative		
HER2-enriched	57	6.3
Basal-like	86	9.5
Unknown	7	0.8
Total	907	100

*HR* hormone receptor; *DCIS* ductal carcinoma in situ

The present study conformed to the principles of the Declaration of Helsinki, and the institutional review board (IRB) of Kyushu University Hospital approved the study (approval no. 30-230). Before surgery, patients provided comprehensive written consent, which stated that the medical information could be used for research purposes.

### Statistical analysis

Progression-free survival (PFS) was assessed for the following four categories: onset at age 45 years or younger; triple-negative breast cancer at age 60 years or younger; two or more primary breast cancers; and a history of ovarian, third-degree relatives with breast or ovarian cancer. Survival curves were generated using the Kaplan–Meier method and were compared using the log-rank test. A *P* value < 0.05 was considered statistically significant. Statistical analysis was carried out using GraphPad Prism version 7.0 (GraphPad Software, Inc. San Diego, CA, USA).

## Results

The distribution of patients who met each condition is summarized in Table 2. Overall, 372 patients (43%) were eligible for *BRCA1/2* genetic testing under the new insurance coverage. The most common category was family history of breast or ovarian cancer within third-degree relatives (*n* = 202/868, 23%). Family history is defined according to the NCCN guidelines® of Genetic/familial high-risk assessment: breast, ovarian, and pancreatic, version 2.2021 [15]. Patients categorized with one condition only were the most frequent (276/868, 32%). Ninety patients (10%) were categorized with two conditions and six (0.7%) were categorized with three conditions. None of the patients were categorized with more than four conditions.

**Table 2:** Distribution of patients who met the new criteria

Category	Number of patients (%)
Onset before age 45 years	153 (18)
Family history of breast or ovarian cancer within the third-degree relatives	202 (23)
Onset before age 60 years with TNBC	48 (6)
Two or more primary breast cancers	64 (7)
Synchronous bilateral breast cancer	35 (9)
Metachronous bilateral breast cancer	27 (7)
Metachronous ipsilateral breast cancer	2 (0.5)
Medical history of ovarian cancer	5 (0.6)
Male breast cancer	1 (0.1)
Above total	372 (43)
Number of applicable categories	
1	276 (74)
2	90 (24)
3	6 (2)
All patients	868 (100)

*TNBC* triple-negative breast cancer

**Fig. 2:**
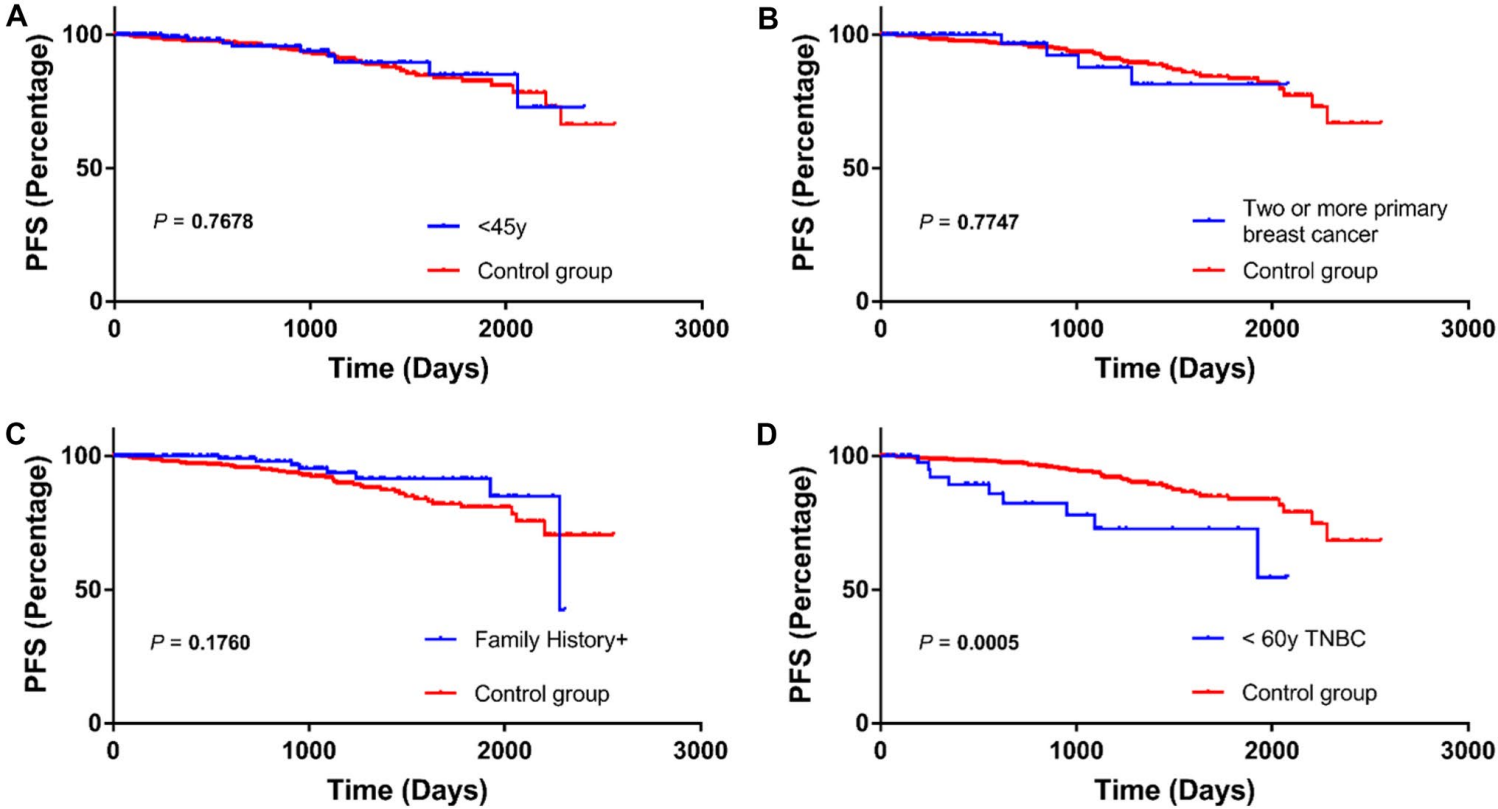
Assessment of progression-free survival (PFS) in four categories comparing the applicable group and control group. < 45y, onset at age 45 years or younger: family history, a history of ovarian, third-degree relatives with breast or ovarian cancer: < 60y TNBC, triple-negative breast cancer at age 60 years or younger

As shown in Fig. 2, there were no significant differences in PFS among the three subgroups: onset at age 45 years or younger (Fig. 2A), two or more primary breast cancers (Fig. 2B), and a history of ovarian, third-degree relative with breast or ovarian cancer (Fig. 2C). The PFS was poor with a statistically significant difference in the subgroup of triple-negative breast cancer at age 60 years or younger, compared to the other patients (*P* = 0.0005; Fig. 2D).

Between July 2018 and September 2021, 105 breast cancer patients underwent *BRCA1/2* genetic testing in our department. Of the 105 patients, 42 were included in the database during this study period. The number of patients with TNBC under 60 years old was 11 (26%) out of the 42 patients. Pathogenic variants were found in 4 patients (9.5%). Three patients of them met three criteria: family history, TNBC younger than 60 years, and onset younger than 45 years. One patient was suspected HBOC as a secondary finding by FoundationOne® CDx. Two patients with pathogenic variants developed distant recurrence (disease-free survival: 951 and 1093 days).

## Discussion

According to our database, 43% of 868 patients were potentially eligible for the BRACAnalysis® testing under the new public insurance criteria. 6.4% of patients that met the new one had ‘HER2-positive’ disease. Olaparib is indicated for metastatic or recurrent ‘HER2-negative’ breast cancer, with which patients are candidates for BRACAnalysis® testing as a CDx for it. Therefore, breast cancer patients despite HER2 status whenever they match the criteria can receive genetic testing with public insurance now. It is known that proportion of *BRCA1/2* mutation career is 2.3% in patients with HER2-positive breast cancer [16].

In this study, the most common category was family history of breast or ovarian cancer within a third-degree relative (23%). Third-degree relatives including cousins, great-grandparents, grandaunts, and granduncles share 12.5% of genetic information with the proband. Thus, family history provides important information for hereditary diseases and must be carefully recorded. Therefore, the information regarding family history is supposed to be obtained from questionnaire that patients filled out, which is stored in our medical record and can be checked by genetic counselors. Once diagnosed as pathogenic or likely pathogenic *BRCA1/2* mutation, it is necessary to carefully consider each treatment plan including adjuvant chemotherapy, prophylactic surgery, reconstructive surgery, and when to perform them. In our hospital, the HBOC team has been established in early 2020, consisting of various departments including Breast Surgery and Oncology, Obstetrics and Gynecology, Plastic Surgery, Pathology and Radiology, as well as Clinical Genetics. We regularly hold conferences where we share information about patients and their family, and discuss risk-reducing surgery and surveillance. The NCCN guidelines suggest the recommended age for RRSO is 35–40 years for *BRCA1* mutations and 40–45 years for *BRCA2* mutations [15], because there is no effective surveillance for ovarian cancer. RRSO should be performed as early as possible at the patient’s request, even if the patient is older than the recommended age. Meanwhile, at present, if unaffected family members of the proband want to receive genetic testing, genetic counseling, surveillance, or risk-reducing surgery, they are supposed to do so at their own expense, which is one of important issues to resolve because of financial burden. In addition, although mutations in *BRCA1/2* increase the risk of prostate cancer [17] and pancreatic cancer [18], surveillance for these cancers has not been defined.

Less frequent susceptibility genes, other than *BRCA1/2*, have been reported for breast cancer. Mutations in the *TP53* (Li-Fraumeni syndrome), *PTEN* (Cowden syndrome), and *CDH1* (hereditary diffuse gastric carcinoma) genes also increase the risk of developing breast cancer [15]. Therefore, even if a negative result with *BRCA1/2* genetic testing cannot rule out hereditary breast cancers, other genetic testing or multi-gene panels may be considered when the medical or family history strongly suggests hereditary cancers. In Japan, a few facilities offer multi-gene panel testing at clients’ own expense, but it is not common.

In December 2020, PARP inhibitor olaparib was indicated for pancreatic cancer in Japan based on the POLO trial [19] and for prostatic cancer based on the PROfound trial [20]. Ovarian cancer treatment added maintenance therapy after initial bevacizumab-containing chemotherapy for cases of ovarian cancer with a defect in homologous recombination repair. BRACAnalysis® is the only accepted companion diagnostic test for olaparib in breast and pancreatic cancer. BRACAnalysis® and FoundationOne® CDx (Foundation Medicine, Inc., Cambridge, MA, USA) for prostate cancer, and BRACAnalysis® and Myriad myChoice® CDx for ovarian cancer are available as companion diagnostic tests. Myriad myChoice® CDx detects the defects in homologous recombination repair and *BRCA1*/*2* mutations by evaluating genomic instability in genomic DNA extracted from tumor tissues.

This study showed that the PFS was significantly poor in the patients with triple-negative breast cancer at age 60 years or younger, compared to the other patients (*P* = 0.0005; Fig. 2D). Currently, the OlympiA trial [21], a multicenter phase III trial to compare the efficacy and safety of olaparib with placebo as adjuvant therapy in patients with high-risk early stage breast cancer with *BRCA1/2* mutations and HER2-negative breast cancer who have completed definitive local treatment and neoadjuvant or adjuvant chemotherapy is underway [21]. Results from the OlympiA trial showed a statistically significant improvement in invasive disease-free survival in the olaparib treatment group [22]. However, there was no significant difference in overall survival between the two groups during the current observation period, though further follow-up is awaited. The positive results of the OlympiA trial suggest that olaparib may be indicated for adjuvant therapy in the near future. If so, patients with TNBC could overcome poor PFS. Thus, diagnosis and treatment associated with HBOC are changing rapidly and complicatedly, regardless of tumor histology.

In addition, it is important to consider VUS related to the increase of genetic testing. In a study of VUS using various genetic tests and multi-gene panels, 24.9% of all VUS were reclassified [23]. In some cases, medical management had changed. According to a study by Esterling et al. on variant classification and reclassification over a 20-year period, 82.1% of reclassified variants were downgraded from VUS to benign/likely benign, whereas 17.9% were upgraded [24]. Based on data from Myriad Genetics, the VUS rate of *BRCA1/2* genetic testing in Asians has declined over time (14.4% in 2005; 8.3% in 2010; 5.7% in 2015; and 4.6% in 2020) [25]. The VUS rate in Japanese was 3.3%–3.4% from 2019 to 2020, which is much lower than the rate of 4.6% for all Asians. The results of genetic testing are important in determining the medical management of patients, and it is necessary to establish a system that enables continuous contact with clients who have received the results of VUS.

This study had some limitations. First, potential patients were identified, but it was not possible to follow up whether they received testing. Because the number of *BRCA1/2* genetic testing performed in our department so small before the approval of BRACAnalysis® for HBOC in primary breast cancers, we did not have enough data to discuss the association between the presence of *BRCA1/2* mutation carrier and their prognosis. Our results showed that TNBC under 60 years of age had a significantly worse PFS, and we investigated prognostic implication of the BRCA mutation positivity in TNBC patients from previous studies and summarized the previous studies as Table 3. These previous studies show that *BRCA1/2* mutation carrier is not a poor prognostic factor in TNBC patients, but it may not be the case in Japan, because medical access to surveillance and prophylactic operation was not available before March 2020. In this regard, the HBOC consortium, which is one of the largest organization for HBOC registry in Japan, has not yet reported the relationship between *BRCA1/2* mutation and prognosis, and it is still unclear.

**Table 3:** Prognostic implication of *BRCA* mutation positive in TNBC patients

Study (authors)	Year	Country	Total cases	*BRCA1*-positive cases (%)	*BRCA2*-positive cases (%)	Prognostic implication between *BRCA 1/2* carriers and non-carriers	Reference
Lee et al.	2011	USA	117	46 (39.3)	–	Adjusting for age and stage, ns in OS (*BRCA1* mutation)	[26]
Bayraktar et al.	2011	USA	227	114 (50)		ns in OS	[27]
Baretta et al.	2016	Italy	105,220 (review)	3588 (3.4)		Better OS in *BRCA* mutation carriers	[8]
Copson et al.	2018	UK	2733	201 (7.4)	137 (5.0)	ns in OS	[9]
Yadav et al.	2018	USA	863	72 (27.0)		ns in OS and RFS	[28]
Ryu et al.	2019	Korea	1628	97 (9.7)	35 (3.5)	ns in OS and DFS	[29]
Pogoda et al.	2020	Poland	502	29 (5.7)	1 (0.1)	ns in OS and RFS (*BRCA1* mutation)	[30]

*TNBC* triple-negative breast cancer; *ns* no significant difference; *OS* overall survival; *RSF* recurrence-free survival; *DFS* disease-free survival

Moreover, another limitation of our study is that the results were only collected from a single institution, and the population may have a bias. Therefore, we believe that our results further strengthen our expectation that the increasing number of *BRCA1/2* genetic testing will reveal the association between *BRCA1/2* mutation and prognosis in the Japanese population and will improve the prognosis for *BRCA1/2* mutation carrier. Therefore, continuous investigation is required.

## Conclusion

The use of genetic medicine is accelerating in Japan, probably because patients with suspected HBOC were eligible for genetic testing by BRACAnalysis®, which was covered by public insurance. Therefore, it would be essential to establish a system for genetic medicine, including counseling and support for patients and their family.

## Supplementary Information

Below is the link to the electronic supplementary material.

Supplementary file1 (DOCX 15 KB)
