# Supplementary material for: Genetic medicine is accelerating in Japan
Source: Breast Cancer. 2022 Feb 21;29(4):659–65. doi: 10.1007/s12282-022-01342-4 (PMC9225975; doi:10.1007/s12282-022-01342-4)
Supplement: Supplementary file 1 — Supplementary file1 (DOCX 15 KB) [file 12282_2022_1342_MOESM1_ESM.docx]

Supplementary Table1. *BRCA 1/2* genetic testing performed in our department

|  | *BRCA1/2* genetic testing | % |
| --- | --- | --- |
| Number of patients | 105 | 100 |
| Included in the present database | 42 | 42 |
| Not included　in the present database | 63 | 58 |
|  |  |  |
| Included in the present database | 42 | 100 |
| *BRCA1/2* pathogenic / likely-pathogenic variant |  |  |
| positive | 4 | 9 |
| negative | 38 | 91 |
|  |  |  |
| 1. Onset before age 45 years | 18 | 43 |
| positive | 3 | 7 |
| negative | 15 | 36 |
| 2. Family history of breast or ovarian cancer within the third-degree relatives | 17 | 40 |
| positive | 3 | 7 |
| negative | 14 | 33 |
| 3. Onset before age 60 years with TNBC | 11 | 26 |
| positive | 3 | 7 |
| negative | 8 | 19 |
| 4. Two or more primary breast cancers | 10 | 24 |
| positive | 0 | 0 |
| negative | 10 | 24 |

TNBC, triple-negative breast cancer
